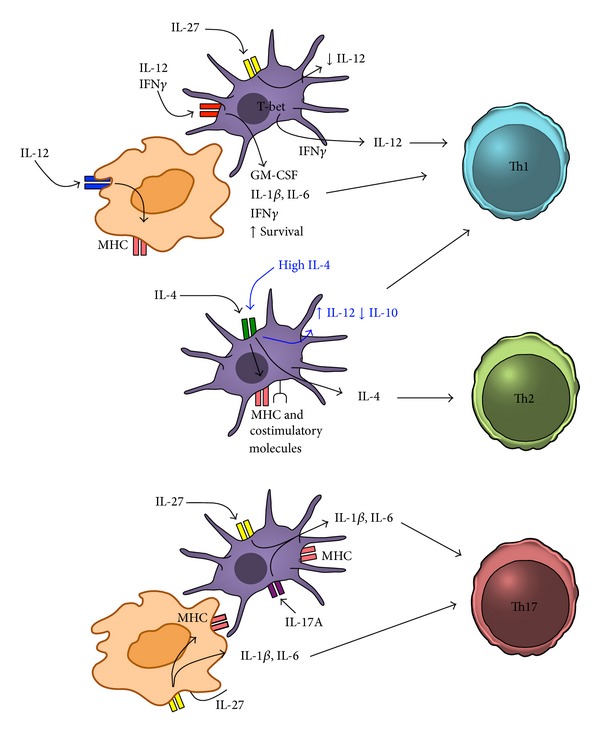# Cellular Factors Targeting APCs to Modulate Adaptive T Cell Immunity

**DOI:** 10.1155/2014/750374

**Published:** 2014-07-14

**Authors:** Anabelle Visperas, Jeongsu Do, Booki Min

**Affiliations:** ^1^Department of Immunology/NB30, Lerner Research Institute, Cleveland Clinic Foundation, 9500 Euclid Avenue, Cleveland, OH 44195, USA; ^2^Department of Molecular Medicine, Lerner College of Medicine of Case Western Reserve University, Cleveland, OH 44195, USA

## Abstract

The fate of adaptive T cell immunity is determined by multiple cellular and molecular factors, among which the cytokine milieu plays the most important role in this process. Depending on the cytokines present during the initial T cell activation, T cells become effector cells that produce different effector molecules and execute adaptive immune functions. Studies thus far have primarily focused on defining how these factors control T cell differentiation by targeting T cells themselves. However, other non-T cells, particularly APCs, also express receptors for the factors and are capable of responding to them. In this review, we will discuss how APCs, by responding to those cytokines, influence T cell differentiation and adaptive immunity.

## 1. Introduction

Naïve CD4 T cells stimulated by cognate antigens presented by professional APCs within the lymphoid tissues undergo clonal expansion and differentiate into distinct lineages of effector/regulatory subsets, orchestrate various adaptive immune responses, and then ultimately mature into memory phenotype cells that either continue to circulate the periphery or migrate into nonlymphoid tissues where they play a role in immune surveillance [1, 2]. Extensive efforts have been made to define cellular and molecular mechanisms that initiate the differentiation processes and to identify the features of each T cell subset. While Th1 type cells producing IFN*γ* are generated in the presence of IL-12, Th2 type cells producing IL-4 and IL-13 are generated in the presence of IL-4. Newly recognized proinflammatory IL-17-producing Th17 type cells are generated in the presence of IL-6 and TGF*β*. On the other hand, Foxp3-expressing inducible regulatory T cells can be generated in the presence of TGF*β*. Other effector/regulatory T cell subsets include Th9 and Tr1 type cells, which produce IL-9 and IL-10, respectively. Although a great number of cellular and molecular pathways to initiate the differentiation programs have already been uncovered, most of studies have heavily focused on those factors that directly target T cells. In this review, we will focus on the roles of factors that control T cell differentiation processes by acting on non-T-cell targets, especially APCs.

## 2. APC Stimulation by IL-12 and IFN

Naïve CD4 T cells activated in the presence of IL-12 become Th1 type effector CD4 T cells and play a key role in eliminating intracellular pathogens and viruses. IL-12 is a heterodimeric protein consisting of the IL-12p35 and IL-12p40 subunits [3], mainly produced by antigen presenting cells (APCs), and its production is greatly enhanced by microbial stimuli such as LPS. IL-12 binds the IL-12 receptor (IL-12R*β*1 and IL-12R*β*2) expressed on naive CD4 T cells, signals through STAT4 upon ligation of the receptor [3, 4], and induces transcription of Ets variant gene 5 (ERM) and T-box 21 [5, 6]. T-box transcription factor* Tbx21*, which encodes T-bet, is a Th1 specific transcription factor controlling the expression of IFN*γ* [7]. Mice deficient in IL-12 display impaired delayed type hypersensitivity responses and succumb to microbial infections such as* Toxoplasma gondii *or* Cryptococcus neoformans* due to the inability to mount Th1 responses [8, 9]. Likewise, T cells deficient in T-bet are unable to differentiate into Th1 cells and T-bet-deficient mice succumb to pathogen infections that are controlled by Th1 cells [7].

T-bet is also expressed by monocytes and myeloid DCs [10]. Indeed, it was reported that T-bet expression in DCs is essential for optimal induction of Th1 immunity in vivo [11]. T-bet-deficient DCs are unable to produce IFN*γ* after stimulation and to induce Th1 differentiation [11]. T-bet expression by DCs is also necessary for the Th2 to Th1 repolarization process that occurs during B7-DC cross-linking on DCs [12].

It is evident that IL-12 directly regulates the function of DCs and macrophages. Monocyte-derived DCs express both subunits of IL-12 receptors [13–15]. Stimulation of DCs by IL-12 induces expression of GM-CSF, IL-1*β*, IL-6, and IFN*γ*, all of which could have a direct impact on T cell differentiation [16]. IL-12 stimulated DCs or macrophages are also capable of presenting an otherwise poorly immunogenic tumor peptide [14, 17]. An inhibitory mechanism of DC production of IL-12 via DC-specific ICAM-3 grabbing nonintegrin receptor 1 (DC-SIGNR1) was also identified. Stimulating DC-SIGNR1 in DCs increases suppressors of cytokine signaling 1 (SOCS1) and lowers IL-12 production [18].

IFN*γ* is the signature cytokine produced by differentiated Th1 type effector cells and regulates IL-18R/IL-12R expression on T cells to prime Th1 differentiation [19]. IFN*γ* also promotes DC maturation and enhances production of cytokines such as IL-1*β* and IL-12 by directly acting on DCs [20]. IFN*γ*R−/− DCs exhibit impaired function to stimulate alloreactive T cells, to drive Th1 differentiation, and to induce efficient antitumor immunity [21]. Similarly, human monocytes in vitro stimulated with cytokine cocktails consisting of TNF*α*, IL-1*β*, IL-6, and prostaglandin E2 produce high levels of IL-10 and low levels of IL-12 [22]. Adding IFN*γ* into the culture condition dramatically increases IL-12 and decreases IL-10 production, thereby enhancing Th1 polarization [22]. IFN*γ* induces IL-27 production in DCs and favors induction of IL-10-producing Tr1 cells, limiting Th17-mediated autoimmune neuroinflammation [23]. The protective role of IFN*γ* is also associated with decreased expression of osteopontin, which is known to have potent proinflammatory function, in DCs [24]. Type I IFN is another potent cytokine similarly influencing adaptive Th17 cell immunity by acting on DCs by regulating expression of IL-27 and osteopontin. IFN*α* acts on DCs, suppresses intracellular translational isoform of osteopontin, known as Opn-i, enhances IL-27 production, and antagonizes Th17 development [25].

We recently reported that IFN*γ* signaling in non-T-cell targets plays a critical role in limiting T cell responses and inflammation in the intestine [26]. Using a model of T-cell-induced colitis, a murine model of human inflammatory bowel disease (IBD) induced by naïve CD4 T cells transferred into immunodeficient recipients, we demonstrated that while transfer of naïve wild type CD4 T cells into Rag−/− recipients induces chronic colitis that develops ~4 weeks after transfer, the same CD4 T cells transferred into IFN*γ*R−/− Rag−/− recipients induce acute fulminant colitis within 7 days after transfer [26]. Unlike above-mentioned studies where IFN*γ* stimulation of DCs alters DC production of cytokines that promote Th1 differentiation, the relative proportion of IFN*γ*-producing CD4 T cell generation in these recipients is similar in the absence of host expression of IFN*γ*R. Instead, the overall expansion of activated T cells (i.e., IFN*γ*- and IL-17-producing CD4 T cells) is dramatically enhanced in this condition [26]. It turned out that IFN*γ* signaling in DCs enhances T cell responses in part by directly controlling survival of DCs. As a result, DCs deficient in IFN*γ*R display enhanced survival in vivo, possibly resulting in prolonged stimulation to T cells [26]. Sercan et al. similarly reported that IFN*γ* signaling in CD11b+ cells controls memory CD8 T cell differentiation [27]. However, a cellular mechanism by which IFN*γ*-mediates memory fate decision remains to be examined.

## 3. APC Stimulation by IL-4

IL-4 is a key cytokine that induces Th2 differentiation by acting on the IL-4 receptors expressed on naïve CD4 T cells [28]. IL-4 signals through STAT6 molecule, activates expression of Th2 associated transcription factors such as GATA3 and c-Maf, and induces expression of Th2 signature cytokines, IL-4, IL-5, and IL-13 [28]. IL-4 exerts its biologic function using two distinct types of IL-4 receptors, type I (IL-4R*α* and *γ*c) and type II (IL-4R*α* and IL-13R*α*1) receptors [29]. The expression of both receptors is different depending on the cell types. While T cells, the major targets of IL-4, express only type I receptors, DCs and macrophages express both receptors [30]. In DCs, differential functions of IL-4 receptors were noted. For example, IL-4 induces DC maturation, upregulating MHCII and costimulatory molecule expression, via type II IL-4R [30]. By contrast, IL-4 also enhances IL-12 production in DCs induced by microbial products, and this function is mediated by the type I IL-4R [30]. IL-4-mediated induction of IL-12 production in DCs is achieved through downregulation of IL-10 [31]. Moreover, IL-4 also induces IL-4 production in DCs [32]. Consistent with these findings, Guenova et al. reported that human DCs differentiated under low-dose IL-4 produce no IL-12 and promote Th2 differentiation, while DCs differentiated under high-dose IL-4 produce large amounts of IL-12 and low IL-10; thereby, Th1 differentiation is preferentially induced [33]. Therefore, IL-4 acting on DCs may influence both Th1 and Th2 differentiation.

Regulatory roles of IL-4 in DC function were recently examined using DC-specific IL-4R*α* deficient animals. Hurdayal et al. reported that IL-4 signaling in DCs plays a key role in mounting protective immune responses using a* Leishmania major* infection model [34]. Susceptibility to* L. major* infection is largely determined by the development of Th2 type responses [35]. Paradoxically, IL-4 administration during early infection enhances IL-12 production by DCs and establishes resistance to* L. major* in susceptible BALB/c mice [36]. CD11c-cre IL-4R*α* ^flox^ mice infected with* L. major* become hypersusceptible to the infection [34].

IL-4-mediated modulation of DC function was also found in other infection models. Infection of IL-4-deficient mice with* Candida albicans* results in impaired development of Th1 immunity [37]. A molecular mechanism underlying the roles of IL-4 in regulating DC production of IL-12 and IL-10 will be a subject of importance especially for vaccine development.

## 4. APC Stimulation by IL-27

IL-27 is a heterodimeric protein consisting of the IL-27p28 and Ebi3 subunits and binds to the IL-27R complexes made of IL-27R*α*/TCCR/WSX-1 and gp130 [38]. IL-27 is primarily produced by APCs activated by IFN*γ*, TLR ligands, or type I IFNs [38]. Both pro- and anti-inflammatory roles of IL-27 have been well documented. For example, Th1 differentiation is greatly enhanced by IL-27, while IL-27 antagonizes Th1, Th2, and Th17 type effector responses in part by inhibiting IL-2 production [39], [38]. IL-27 induces T-bet expression and activates both STAT1 and STAT4, promoting Th1 differentiation [10, 40]. Accordingly, mice deficient in IL-27R*α* − /− infected with intracellular pathogens including* Listeria*,* Leishmania*, and* Mycobacteria* are more susceptible to the infections [41]. Following* Toxoplasma gondii* infection in IL-27R*α* − /− mice, a robust IFN*γ* response is induced and the clearance of the infection is rapidly achieved. However, these mice succumb to uncontrolled immune activation [42], strongly suggesting that IL-27 not only may promote Th1 differentiation, but also may be important to suppress overactive immune responses, possibly by inducing IL-10 by CD4 T cells [43]. IL-27-mediated suppression of Th17 immunity is also supported by the fact that IL-27R*α*2212/− mice develop more severe experimental autoimmune encephalomyelitis (EAE) [44].

Immunoregulatory roles of IL-27 described above are examples of IL-27 action on T cells. However, IL-27 receptors are expressed on multiple cell types, including DCs and macrophages [38]. IL-27 upregulates MHC and TLR4 expression in human monocytes, resulting in increased production of IL-1*β* and IL-6 following LPS stimulation [45]. Alternatively, IL-27 downregulates IL-12 production by activated macrophages in* Mycobacterium tuberculosis* infection models [46]. IL-27 is also capable of inducing B7-H1 expression in DCs, which then display reduced function to stimulate allogeneic T cell responses [47]. IL-27 was recently reported to induce CD39 expression in DCs and interferes with EAE development [48]. CD39 induced by IL-27 decreases the extracellular ATP and downregulates ATP-mediated activation of the NLRP3 inflammasome [48].

By contrast, we recently reported that IL-27 signaling in non-T cells especially macrophages and DCs plays a crucial role in generating proinflammatory Th17 responses [49]. This conclusion is made based on the T-cell-induced colitis model using lymphopenic mice deficient in IL-27R*α*. While IL-27R*α*+ lymphopenic mice that receive naïve CD4 T cells develop chronic colitis associated with colitogenic Th1 and Th17 type effector cell generation, lymphopenic mice deficient in IL-27R*α* are completely protected from the colitis after the T cell transfer. Interestingly, generation of IL-17-producing CD4 T cells is completely abrogated in this condition, while IFN*γ*-producing CD4 T cell generation remains unaltered [49]. To investigate which host cells are responsible for the lack of Th17 differentiation, we next examined DCs and macrophage expression of cytokines promoting Th17 differentiation, namely, IL-1*β* and IL-6. Indeed, APCs from IL-27R*α* − /− mice failed to produce IL-1*β* and IL-6. Therefore, it seems that IL-27 produced by activated APCs acts on the same or neighboring APCs to increase production of Th17-promoting cytokines. Unlike above-mentioned studies, we were not able to find immunosuppressive roles of IL-27 in DCs. It will be important to identify cellular and molecular pathways of IL-27 leading to immunostimulatory or immunosuppressive function of DCs.

## 5. APC Stimulation by Other Cytokines

### 5.1. IL-21

IL-21 is a cytokine produced by activated CD4 T cells and NK cells. Previous studies demonstrated a key role for IL-21 in Th17 differentiation, when acting with TGF*β*, as a redundant cytokine to IL-6 [50]. IL-21 also amplifies Th17 differentiation in concert with IL-23 [50–52]. IL-21 is known to have a pleiotropic role by controlling multiple cell functions. IL-21 inhibits DC function to mediate T cell activation [53]. Thus, IL-21-treated DCs are unable to induce CD8 T cell proliferation and contact hypersensitivity responses [53]. It was also reported that IL-21 induces granzyme B expression in human plasmacytoid DCs, which then partially impairs pDC function to stimulate T cell proliferation [54]. Type I diabetes susceptibility locus known as insulin-dependent diabetes susceptibility 3 (*Idd3*) encodes cytokine gene IL-21 and regulates diabetes [55]. It was demonstrated that APCs from diabetes-susceptible NOD and diabetes-resistant NOD.* Idd3* mice differentially support diabetogenic Th17 immunity and that IL-21 signaling in APCs plays a critical role in regulating the Th17-promoting APC functions [55].

### 5.2. IL-17

IL-17 is the signature cytokine produced by Th17 cells. Many Th17 cells produce IL-17A and IL-17F. IL-17 plays a protective role in host defense against extracellular pathogens and fungus especially at the epithelial and mucosal surface [56]. IL-17 promotes the generation of inflammatory cytokines and chemokines, which attract neutrophils and macrophages to the sites [57, 58]. IL-17-responding targets cells include nonhematopoietic cells including fibroblasts and epithelial cells, as well as macrophages and neutrophils. IL-17 also acts on DCs [59]. IL-17A but not IL-17F stimulates bone marrow derived DCs to secrete more IL-12, IL-6, and IL-1*β* [59]. IL-17A also upregulates MHCI expression in DCs [59]. IL-17 stimulation in DCs enhances cross-priming function for CD8 T cells during* Listeria* infection [59], although whether it also affects CD4 T cell responses remains to be examined.

## 6. Conclusion

Activated APCs are the main sources of cytokines capable of influencing adaptive T cell responses. As discussed in this review, there is evidence that APCs are also potent target cells of these cytokines, and the stimulation of APCs might have equally important roles in generating different effector immunity (Figure 1). Therefore, many cytokines will affect T cell immunity by acting on the T cells in concert with its targeting on APCs. In order to dissect the pathways in vivo, utilizing animal models with a cell type specific deletion of cytokine receptors will be an ideal approach. Endeavor to understand the pathways has already begun.

## Figures and Tables

**Figure 1:**